# Bio-inspired polymer array vapor sensor with dual signals of fluorescence intensity and wavelength shift

**DOI:** 10.3389/fbioe.2022.1058404

**Published:** 2022-10-31

**Authors:** Zhihao Zhao, Yinghao Ge, Lingyun Xu, Xiaohan Sun, Jing Zuo, Zhenglin Wang, Hongyang Liu, Xiangyu Jiang, Dong Wang

**Affiliations:** ^1^ Research Institute of Frontier Science, Beihang University, Beijing, China; ^2^ Department of Materials Physics and Chemistry, School of Materials Science and Engineering, University of Science and Technology Beijing, Beijing, China; ^3^ Chinese Acad Sci, Tech Institute Phys & Chem, CAS Key Lab Bioinspired Mat & Interfacial Sci, Beijing, China; ^4^ School of Future Technology, University of Chinese Academy of Sciences, Beijing, China

**Keywords:** aggregation-induced emission, one-dimensionalization, organic vapor sensor arrays, fluorescent intensity, wavelength shift

## Abstract

Organic vapor sensors based on polymer owing to their tunable molecular structures and designable functions have attracted considerable research interest. However, detecting multiple organic vapors with high accuracy and a low detection limit is still challenging. Herein, inspired by the mammalian olfactory recognition system, organic vapor sensors based on one-dimensional microfilament array structures with a wide range of sensing gases are demonstrated. By introducing aggregation-induced emission (AIE) molecules, sensors possess dual-optical sensing mechanisms of variation in fluorescence intensity and wavelength. By virtue of the synergistic effects of dual signals, superb accuracy and incredibly low detection limit are achieved for identifying analytes. In particular, the polymer/AIE microfilament array can detect acetone vapor down to 0.03% of saturated vapor pressure. In the saturated vapor of acetone, the fluorescence intensity of the sensor arrays was reduced by 53.7%, while the fluorescence wavelength was red-shifted by 21 nm. Combined with the principal component analysis (PCA) algorithm, the polymer/AIE molecular sensor arrays accomplished the classification and identification of acetone, ethanol, methylene chloride, toluene, and benzene. This bioinspired approach with dual sensing signals may broaden practical applications to high-performance gas sensors for precise molecular detection.

## 1 Introduction

Nowadays, the electronic nose has been intensively studied, and a variety of array sensors have been developed (Lipatov et al., 2013; Rochat and Swager, 2014; Zhang et al., 2017) to detect metal ions, toxic gases, flammable and explosive gases (Zhu Q. et al., 2017), volatile organic compounds and analyze complex mixtures such as fish freshness (Liu et al., 2022) and wine (Han et al., 2022). Traditional chemical sensors use a “lock and key” design strategy to identify analytes, relying on a well-designed highly selective receptor. Such methods are suitable for identifying specific target compounds when the ambient atmosphere is disturbed. However, if the analyte changes, another highly selective receptor must be redesigned, which is very time-consuming and laborious. Therefore, it is still an urgent challenge to develop a universal, effective, wide detection range and low detection limit organic vapor sensor.

Inspired by the superior performance of biological olfactory systems, sensor arrays have been presented to complement traditional chemical sensing methods (Persaud and Dodd, 1982; Li et al., 2019). Mammals perceive the external environment by using the smell system to find food, identify territories and determine enemies. The mammalian olfactory system contains approximately 1,000 different olfactory receptor genes (Mombaerts, 1999; Menini et al., 2004; Buck, 2005). The same receptor can respond to a wide range of analytes but cannot differentiate an analyte accurately. To distinguish specific odors, cross-reactive sensor arrays are formed by a large number of olfactory receptors without specific recognition. Owing to these its-for-recognition arrays, humans can distinguish more than 1 trillion olfactory stimuli (Bushdid et al., 2014). In artificial sensor arrays, which are composed of a series of sensing units without specific recognition capabilities, each element of the sensor array is capable of responding to diverse chemicals or classes of chemicals and do not need to be individually high-selective to any given analyte.

The capillary-bridge-mediated assembly (CBMA) method is an emerging technique to self-assemble materials into a directionally arranged micron filament structure (Feng et al., 2017; Feng et al., 2018; Chen et al., 2021). This ordered quasi-one-dimensional structure allows the sensor to obtain a larger specific surface area than conventional thin-film-like sensors, which will result in better sensor performance (Su et al., 2012; Chen et al., 2013).

Fluorescence sensor arrays, due to their high sensitivity, no need of reference systems, diverse output signals, and ability to image (McDonagh et al., 2008; Wu et al., 2015), have become the research hotspots in recent years. The existing fluorescence sensor response signals are mainly either fluorescence intensity changes (Jiang et al., 2018; Liu et al., 2018; Stevens et al., 2020) or spectral wavelength shifts (Zhu J. et al., 2017; Zhu et al., 2019; Yao et al., 2021). Employing multiple fluorescence response signals for detecting analytes is a feasible strategy to improve the differentiation degree of the sensor arrays (Teo et al., 2009; Koo et al., 2011).

Almost all polymers undergo swelling in the presence of organic vapors. During the swelling process, the macroscopic volume of polymers becomes larger, and the molecular chain gaps subsequently become more spacious. By introducing aggregation-induced emission (AIE) molecules, the volume variant of the polymer can be reflected by the fluorescence signal change (Leung et al., 2014; Mei et al., 2014; Kwok et al., 2015; Gao and Tang, 2017). Different polymers swell to different degrees under the same organic vapor atmosphere, which provides a possibility of designing optical organic vapor sensor arrays with the swelling behavior of macromolecules (Jiang et al., 2018; Jiang et al., 2021).

In this work, one-dimensional arrays containing polymer and AIE molecules have been fabricated by the CBMA method. Based on the polymer swelling principle, organic vapor sensors with two sensing mechanisms of fluorescence intensity change and spectral shift have been obtained. Sensing arrays consisting of four gas sensors with different polymeric substrates were achieved to mimic the olfactory process of mammals. Combined with PCA algorithms, sensor arrays were used to successfully analyze and identify the vapors of five common volatile organic solvents: acetone, ethanol, methylene chloride, benzene, and toluene. Finally, the five different types of organic gases are well separated in the 2D PCA images.

## 2 Materials and methods

### 2.1 Materials

Four commercially available polymers, including polystyrene (PS), polyethersulfone (PES), polyvinylpyrrolidone (PVP), and polymethyl methacrylate (PMMA), were purchased from Sinopharm Chemical Reagent Co., Ltd., China and used without purification. 2-[[4'-(Diphenylamino)[1,1′-biphenyl]-4-yl]methylene]propanedinitrile (TPMN) molecules were provided by AIE Institute, China. The photoresist (KMP CP4800) was supplied by Kempur Microelectronics Inc. China. Organic solvents were bought from Shanghai Aladdin Reagent Co. Ltd. Shanghai, China.

### 2.2 Production of silicon column template

Line-structured silicon column templates were prepared in the following steps. Silicon wafers (100 mm diameter, P-doped, <100> orientation, 400 µm thick) were structured by photolithography and deep reactive ion etching using a direct laser writing device. Then, periodic micropillar structured substrates with a gap of 5 µm between two adjacent columns, a width of 2 μm, and a height of 15 µm were fabricated. The photoresist was removed by cleaning in a plasma cleaner for 20 min.

### 2.3 Asymmetric wettability modification of silicon micropillar templates

The template was rinsed with deionized water and acetone in turn, immersed in ethanol for about 5 min, and blown dry with dry nitrogen. Meanwhile, the slides were cleaned with ethanol. The photoresist was spin-coated on the slide using a spin coater (LEBOscience, KW-4A, China). The side with photoresist film was pressed onto the microcolumn template, on which a 10 g weight was placed and left for 20 s. After the silicon template was peeled off, the photoresist was irradiated with 365 nm UV light for 3 min to cure the photoresist. As a result, the top of the silicon column was protected by the photoresist film, while the sidewalls and microstructure gaps of the silicon column were exposed to air. The silicon template was then fumigated in a FAS atmosphere at 60°C for 12 h. In the process, the sidewalls of the silicon micro-pillars and their interstitial surfaces were modified by FAS molecules with low surface energy. The protective film on the top of the silica column was removed to obtain an asymmetric wettability template with a hydrophilic top and hydrophobic side walls, as shown in Supplementary Figure S1.

### 2.4 Preparation of polymer/AIE molecular microfilament arrays

First, a certain amount of PS and TPMN (mass ratio of 10:1) was dissolved in dichloromethane in various concentrations of 1 g/L, 5 g/L, and 10 g/L. Then, the highly aligned 1D arrays of polymer/AIE were prepared by the CBMA method using a microcolumn silicon template with a width of 2 μm, spacing of 5 μm, and a height of 15 µm. The schematic diagram is shown in Supplementary Figure S2. 10 µL of the polymer/AIE molecules solution was dropped onto the template and covered with a clean quartz sheet to construct a sandwich structure, and then placed in a fume hood for 24 h to slowly evaporate the solvent at room temperature. It was transferred to a vacuum drying oven at 60°C for 2 h to evaporate any possible residual organic solvent. Eventually, highly aligned polymer/AIE molecular microfilament arrays were obtained on quartz sheets.

### 2.5 Characterization

Bright-field microscopy images were obtained by light microscopy (Olympus, BX53, Japan) and a charge-coupled device (CCD) camera (Olympus, DP80, Japan) under a daylight light source. Meanwhile, fluorescence images were obtained under the excitation of a mercury lamp light source. The morphology of the assembled arrays was observed using a cold-field emission scanning electron microscope (SEM, Hitachi, SU8010, Japan) with an accelerating voltage of 10.0 kV. The dimensions of microstrips arrays were measured *via* a confocal microscope (Olympus, OLS-45-SAF, Japan) with atomic force microscopy (AFM) mode.

### 2.6 Organic vapor sensing measurement

A micro sampler is used to inject a quantitative amount of organic solvent into a Teflon gas bag containing nitrogen to obtain a specific concentration of organic vapor. The organic vapor was continuously blown into the quartz hood so that the sample was in full contact with the organic vapor. Subsequently, the fluorescence spectra of polymer/AIE molecular microfilament arrays in different gas environments were obtained using a fluorescence spectrometer (Shimadzu, RF-5301pc, Japan) with an excitation wavelength of 480 nm. The changes in fluorescence intensity and peak shifts of polymer/AIE molecules microfilament arrays in different gas atmospheres were then obtained.

## 3 Results

### 3.1 Fabrication of 1D polymers/AIE molecules arrays

**FIGURE 1:**
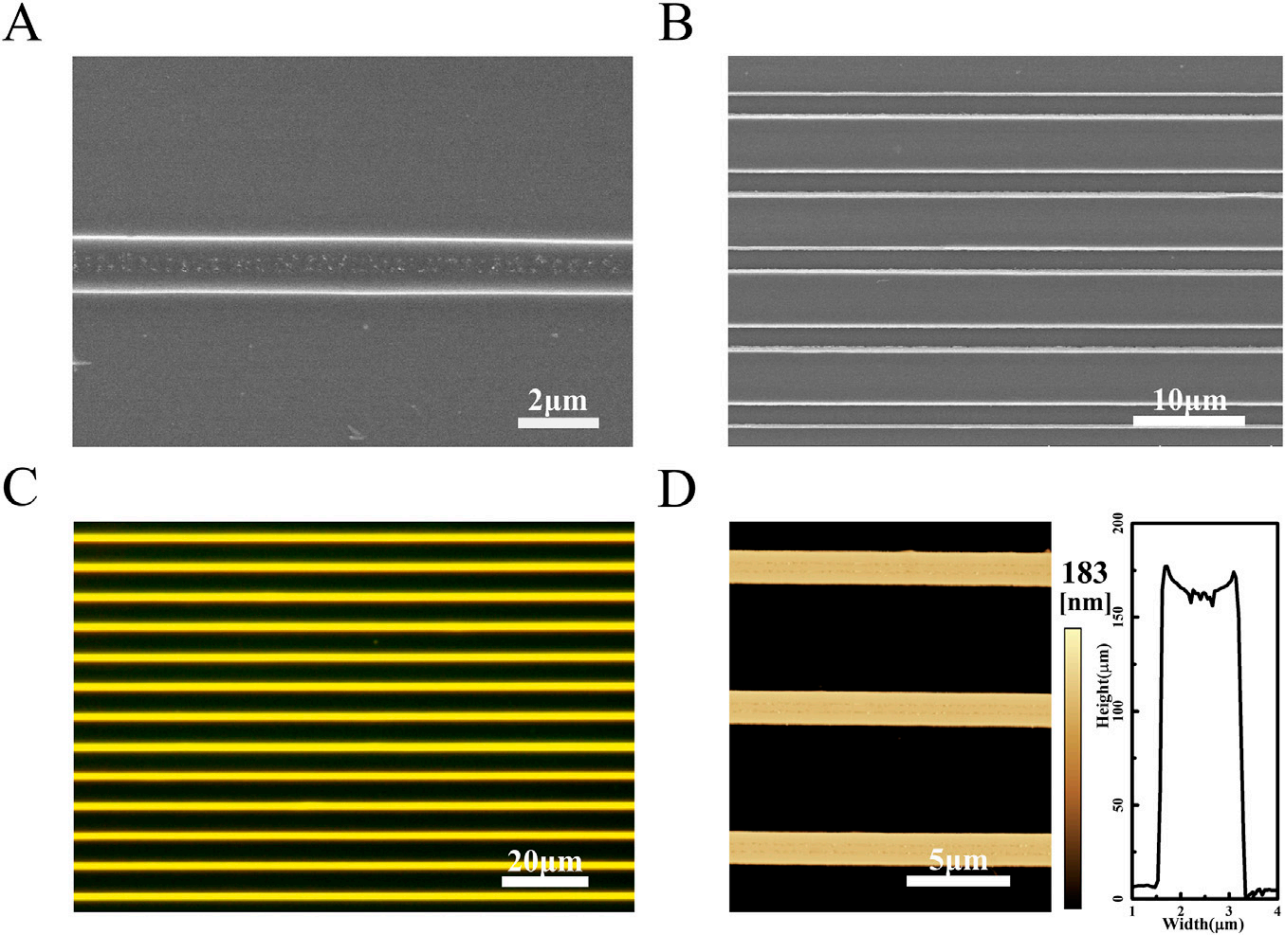
**(A)** SEM image of a single PS/TPMN molecular microfilament array. The width of the microfilaments is 1.41 ± 0.02 µm, and they have flat and smooth edges **(B)** Multiple PS/TPMN molecular microfilament arrays arrange neatly. The spacing between two adjacent microfilaments is 5.44 ± 0.11 µm. The microfilaments are equally spaced and arranged in parallel; **(C)** Dark-field microscopy photograph of the microfilament array with uniform yellow fluorescence emission from the microfilaments; **(D)** AFM photograph of the microfilament array and cross-sectional of the microfilament array. The microfilaments are uniform in height with a height of 183 nm.

In order to explore the optimal concentration for the preparation of micron filament arrays by the CBMA method, polymer/AIE molecules solution at different concentrations was formulated. SEM images show that the micron filaments became increasingly smooth as the solution concentration increased (Supplementary Figure S3). At the solution concentration of 10 g/L, the prepared micron filaments possess straight edges and regular arrangement. Therefore, the optimal solution concentration is determined to be 10 g/L. Compared with unmodified templates, arrays prepared using the asymmetric-wettability modification have a larger range of highly aligned arrangements (Supplementary Figure S4). For the unmodified microcolumn templates, the solution contraction is subjected to less induced force and more random, causing aligned microfilament arrays unsuccessfully assembled. As shown in Figure 1A, the obtained polymer/AIE molecules arrays present flat edges and highly parallel arrangements. In the dark-field microscopy image, the sample emits a uniform and bright orange-yellow light, which indicates that the AIE molecules are uniformly distributed in the polymer microfilaments (Figure 1C). To further identify the dimensions of micro stripes, AFM was performed. AFM images demonstrate that the micro stripes in the arrays are uniform in height and similar in width (Figure 1D).

#### 3.2 Sensing performances of 1D arrays of polymer/AIE molecules

**FIGURE 2:**
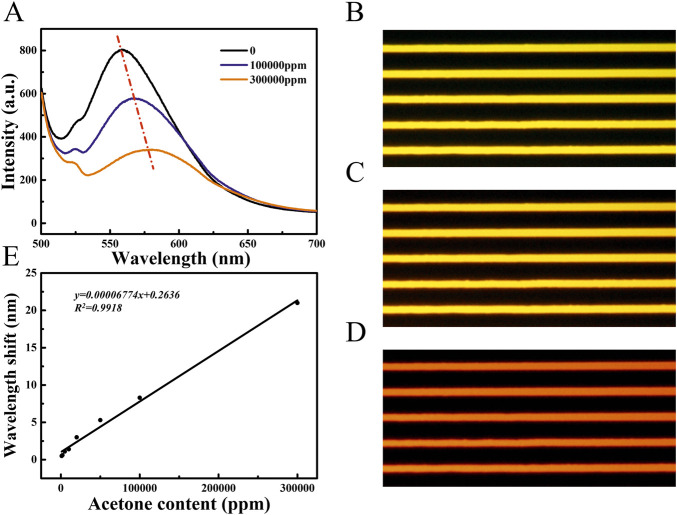
**(A)** Fluorescence spectra of PS/TPMN samples in the gaseous atmosphere at 0, 100000 ppm, and 300000 ppm acetone concentrations. Compared with the acetone concentration of 0, the PS/TPMN samples were red-shifted by 8.3 nm and 21.0 nm in the atmosphere containing 100,000 ppm and 300,000 ppm of acetone vapor, respectively, and the fluorescence intensity was reduced by 22.89 ± 0.81%, and 53.72 ± 1.66%, respectively **(B)** Fluorescence photographs of PS/TPMN samples in the air; **(C)** Fluorescence photographs of PS/TPMN samples in acetone atmosphere at 100000 ppm **(D)** Fluorescence photographs of PS/TPMN samples in 300000 ppm acetone atmosphere; **(E)** Fluorescence photographs of PS/TPMN samples red-shifted in acetone vapor atmosphere at 1,000–300000 ppm, and the degree of red-shift showed a good linear relationship with acetone vapor concentration.

As shown in Figure 2A, the response of PS/TPMN arrays to acetone vapor was demonstrated. It can be seen that the PS/TPMN sample generates a significant response to acetone vapor. To begin with, its fluorescence spectrum was tested in a laboratory air atmosphere, and the sample emitted the strongest fluorescence at 480 nm excitation light. The samples were then placed in an acetone vapor atmosphere at 100,000 ppm and 300,000 ppm, and the fluorescence intensity was found to be significantly lower. Compared with the fluorescence intensity in the air, the fluorescence intensity decreased by 22.9% and 53.7% in the acetone atmosphere at 100,000 ppm and 300,000 ppm, respectively (Figure 2A). To explain the underlying mechanism of vapor sensing, confocal images of PS/TPMN samples in the air atmosphere and 300000 ppm acetone vapor were taken. The width and height were expanded by 7.18 ± 1.17% and 8.66 ± 0.87% under the acetone atmosphere, respectively (Supplementary Figure S5), which verified that the decrease in fluorescence intensity of the optical gas sensors is due to the swelling effect after adsorption of organic vapors.

The shift in peak position can also be observed along with the change in fluorescence intensity with acetone concentration increased in the ambient atmosphere at the fluorescence spectrum. Compared with the central wavelength of fluorescence in air, the fluorescence intensity was red-shifted by 8.3 nm and 21.0 nm in the acetone atmosphere at 100,000 ppm and 300,000 ppm, respectively. Meanwhile, a change in fluorescence color from yellow to orange and red can be observed in the fluorescence micrographs (Figures 2B–D). Afterward, a test of acetone concentration on the fluorescence wavelength change of PS/TPMN arrays was conducted, and no significant wavelength change was observed at less than 1,000 ppm. Compared with the peak position in the air atmosphere, the wavelength redshift by 0.5 nm in 1,000 ppm acetone atmosphere. With the acetone concentration increased, a good linear relationship between the wave shift and the acetone concentration in the environment was established (Figure 2E). It indicates that the polymer/AIE molecular microfilament array sensor can complement the gas response by using wave peak shift while detecting organic vapor through fluorescence intensity change.

**FIGURE 3:**
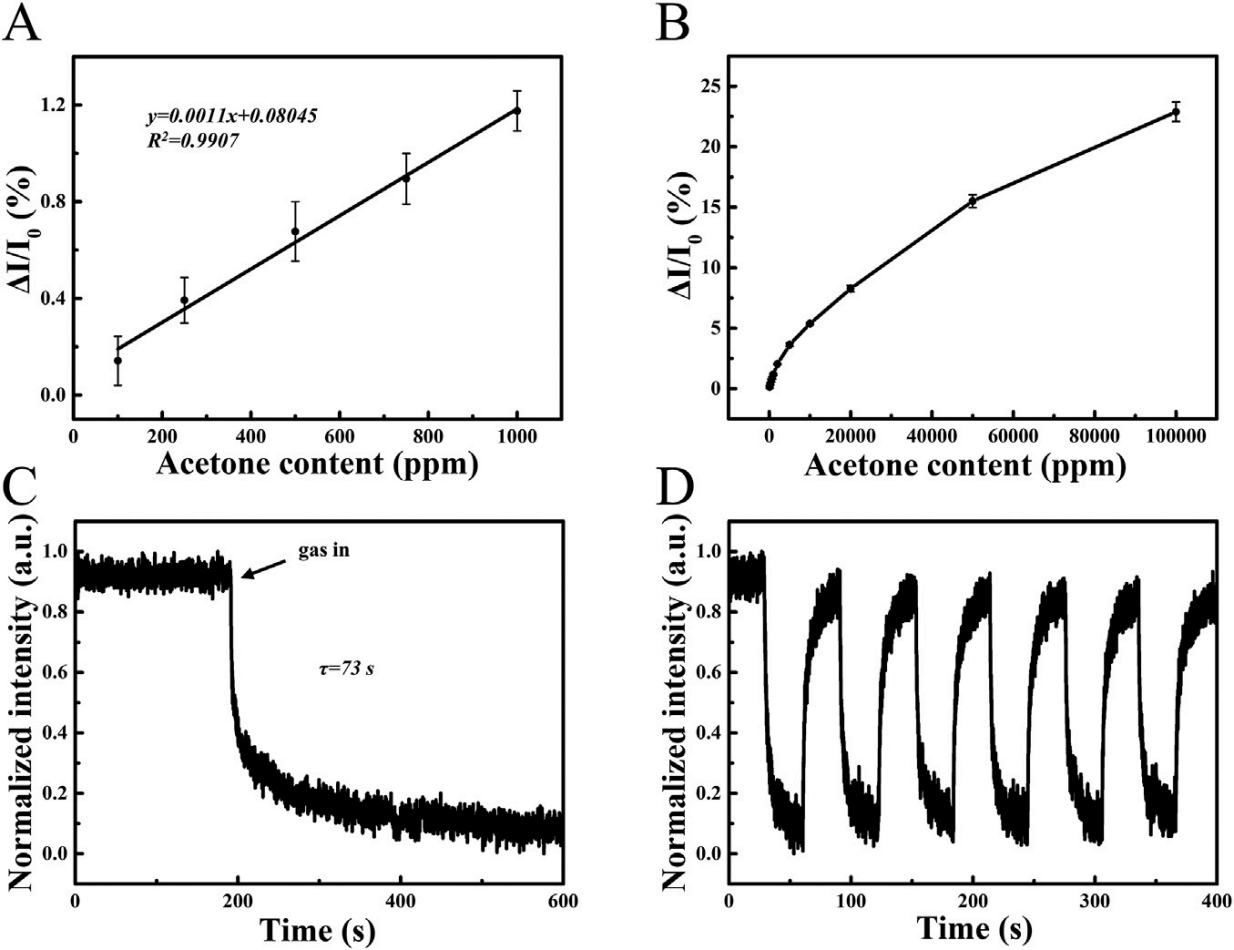
**(A)** Response curves of PS/TPMN samples in acetone vapor at low concentrations. ΔI/I_0_ varies linearly with acetone concentration, demonstrating a good linear dependency with low limit of detection of 100 ppm. Error bars, s.d. (*n* = 10); **(B)** Response curves of PS/TPMN samples in acetone vapor at 100 ppm-100000 ppm. Error bars, s.d. (*n* = 10) **(C)** Single response curves of PS/TPMN samples in acetone vapor at 300000 ppm. The result indicates the sensor response time is 73 s; **(D)** Continuous multiple response curves of PS/TPMN samples in acetone vapor at 300000 ppm. Demonstrates good reproducibility and stability of the sensor.

Fluorescence spectroscopy of PS/TPMN arrays under different concentrations of acetone vapor was performed. The results show that the fluorescence intensity change (ΔI/I_0_) tends to increase with increasing acetone concentration, where I_0_ is defined as the baseline fluorescence intensity of PS/TPMN microfilaments in air, and ΔI indicates the fluorescence intensity difference after and before exposure to acetone vapor. The fluorescence intensity change remained well linear with acetone concentration at less than 1,000 ppm (Figure 3A). With a further increase in acetone concentration, the rising trend of ΔI/I_0_ becomes gradually smaller. Figure 3B gives the trend graph of fluorescence intensity variation for the acetone concentration range of 100 ppm–100000 ppm. As shown in Figures 3A,C sensing response time of 73 s for 300,000 ppm acetone vapor was achieved. In addition, the adsorption-desorption cycles of PS/TPMN arrays were conducted and exhibited reliable stability in the saturated vapor of acetone (Figure 3D).

**FIGURE 4:**
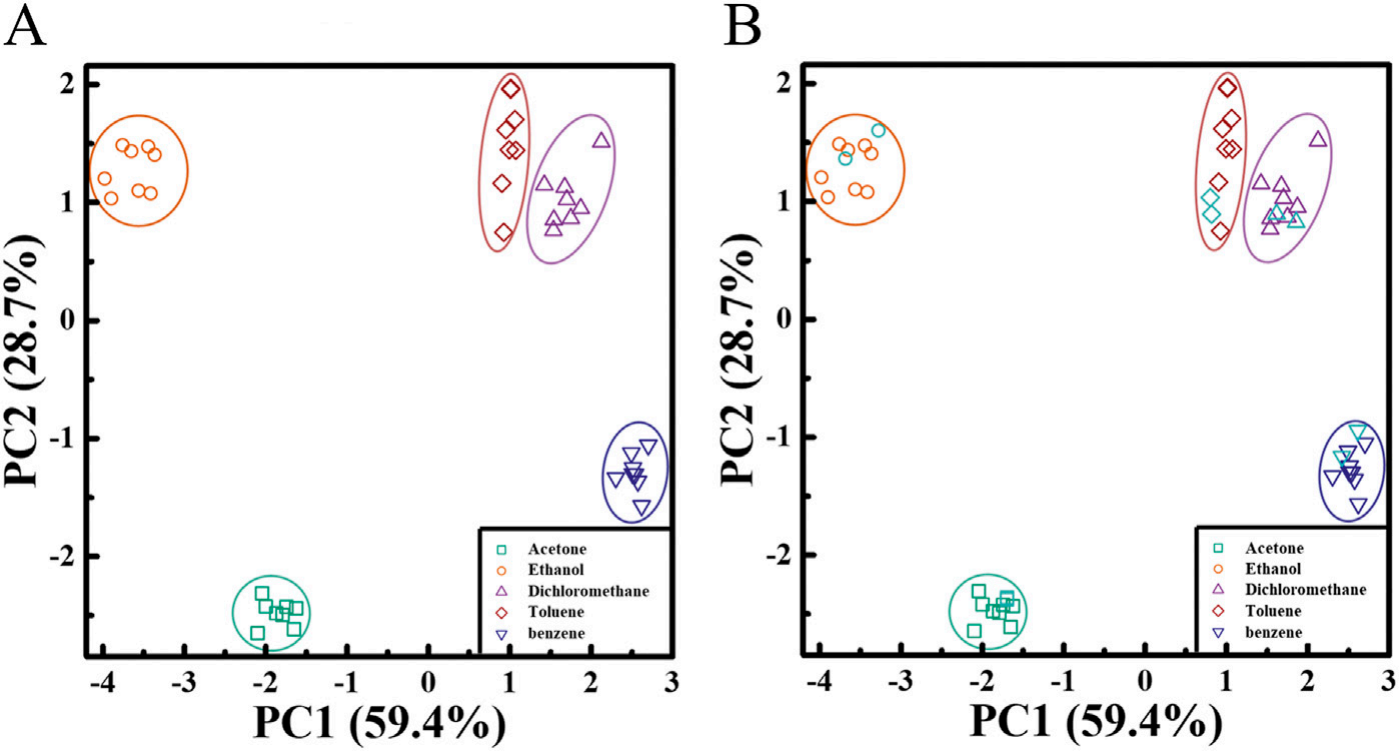
Array-based organic vapor sensing and similar organic vapor identification **(A)** PCA plots, calculated from fluorescence intensity variation data obtained for four polymer/TPMN array sensors during exposure to five organic vapors, as shown in the first two main axes of PC1 and PC2. The four commercial polymers used include PS, PMMA, PES, and PVP, and the AIE molecule is TPMN. The PCA algorithm shows that PC1 and PC2 capture 88.1% of the variance of the data, indicating that the different types of organic vapors are well separated. **(B)** Validation of the proposed classification method. Two additional sets of data from these five organic vapors (sky blue) were measured and evaluated, and whether the sensors could correctly identify organic vapors by their vapor locations in the PCA plot. The results show that our proposed method can classify and identify the different organic vapors well.

Four different polymer/AIE molecule microfilament array sensors were prepared using four different commercial polymers (PS, PES, PVP, and PMMA) in combination with TPMN. Commonly used organic vapors of acetone, ethanol, methylene chloride, toluene, and benzene were detected by these four polymer/TPMN sensor arrays (all at 10,000 ppm vapor concentration). By collecting fluorescence intensity variation data, the different types of organic vapors were classified using the PCA method. The PCA result shows that the first two principal components, PC1, and PC2, could capture 88.1% of the variation in the data, accounting for 59.4% and 28.7% of the variance, respectively (Figure 4A), and the first two principal components were sufficient for classification. The different types of organic vapors were well separated. Two more sets of data were measured for these seven organic vapors and evaluated whether the sensor could correctly identify organic vapors by the location of the vapors in the PCA plot. A five-nearest points estimation method based on the first two principal components was applied to perform the classification. Specifically, the first two principal component scores for new observations were calculated and plotted. Then, for each new observation, it is determined that the observation belongs to the group with the highest weight of the five-nearest points in the training dataset, as shown in Figure 4B (plotted in sky-blue color). The overall results show that our proposed method can well classify and identify different organic vapors with a classification rate of 100%.

## 4 Discussion

1D sensors have a larger specific surface area, compared to thin-film sensors, which can achieve fuller contact between the sensor and the environment. Therefore, one-dimensionalization is a feasible idea to enhance the sensitivity and response speed of sensors. Significant progress has been made in the one-dimensionalization of materials and 1D gas sensors. However, in the fabrication of the 1D materials-based devices, the 1D materials exhibit disordered arrangements with random positions and orientations. By using the CBMA method, we precisely align PS with TPMN fluorescent molecules into 1D micro stripe arrays.

Owing to the precise alignment of 1D structures, which are only confined at the bottom, the sensing material can be fully exposed to ambient gas. Under organic vapors, the microfilaments can expand not only upward but also to the left and right sides. The confocal microscope results show that the width and height of the dissolved 1D structure become larger by 7.18 ± 1.17% and 8.66 ± 0.87%, respectively. In conclusion, the 1D array structures allow for higher sensitivity and a more intense response of the organic vapor sensor based on the swelling principle. The PS/TPMN microfilament arrays can respond to acetone vapor down to 0.03% of the saturation vapor pressure and maintain good linearity between 100 ppm and 1,000 ppm acetone concentration. The polymer/AIE molecule microfilament arrays exhibit good stability when exposed to saturated acetone vapor. A redshift of the wave peak was observed at acetone concentrations up to 2000 ppm. And the central wavelength of sample fluorescence varied linearly with the organic vapor concentration over the acetone concentration range of 2000 ppm–300000 ppm. The polymer/AIE molecule microfilament array sensor can respond to organic vapor with both intensity change and color change. Compared with single-signal organic vapor sensors, it provides more reliable detection results.

Four different polymer/AIE molecular microfilament array sensors were demonstrated using four commercial polymers in combination with TPMN fluorescent molecules. Based on the PCA results, we successfully classified and identified five common organic volatile gas molecules. Among them, two similar organic gases benzene and toluene were still able to be distinguished completely. It proves that the polymer/AIE molecular microfilament sensor arrays based on the polymer swelling principle have good discriminative ability.

## 5 Conclusion

In conclusion, optical organic vapor sensors have been prepared using the CBMA method, which includes polymer/AIE molecular microfilament arrays with smooth surfaces and uniform dimensions. With the assistance of swelling and AIE effects, obtained sensors can undergo a reversible fluorescence change in an organic vapor atmosphere. Based on the dual-optical sensing response of intensity change and wavelength shift, the optical gas vapor sensors exhibit improved accuracy and broaden detection range. In particular, the fluorescence intensity changes of the PS/TPMN microfilament arrays were observed in acetone vapor from 100 ppm to 100000 ppm, and the fluorescence intensity variation remained well linear with acetone vapor concentration in the low concentration range of 100–1000 ppm. In addition, the fluorescence wavelength shifts of the PS/TPMN microfilament arrays show good linearity in acetone vapor from 1,000 ppm to 300000 ppm. The universality of this technique was verified by adopting four commercial polymers (PS, PES, PVP, and PMMA). Five commonly used organic gases (acetone, ethanol, methylene chloride, toluene, and benzene) were accurately identified by these sensors, especially two similar gases of toluene and benzene. These attractive results demonstrate that polymer arrays with dual optical responses have the potential to be developed into practical and versatile gas sensors in various application fields.

## Data Availability

The original contributions presented in the study are included in the article/Supplementary Material, further inquiries can be directed to the corresponding authors.
