# Supplementary material for: Bio-inspired polymer array vapor sensor with dual signals of fluorescence intensity and wavelength shift
Source: Front Bioeng Biotechnol. 2022 Oct 31;10:1058404. doi: 10.3389/fbioe.2022.1058404 (PMC9659642; doi:10.3389/fbioe.2022.1058404)
Supplement: Supplementary file 1 [file DataSheet1.docx]

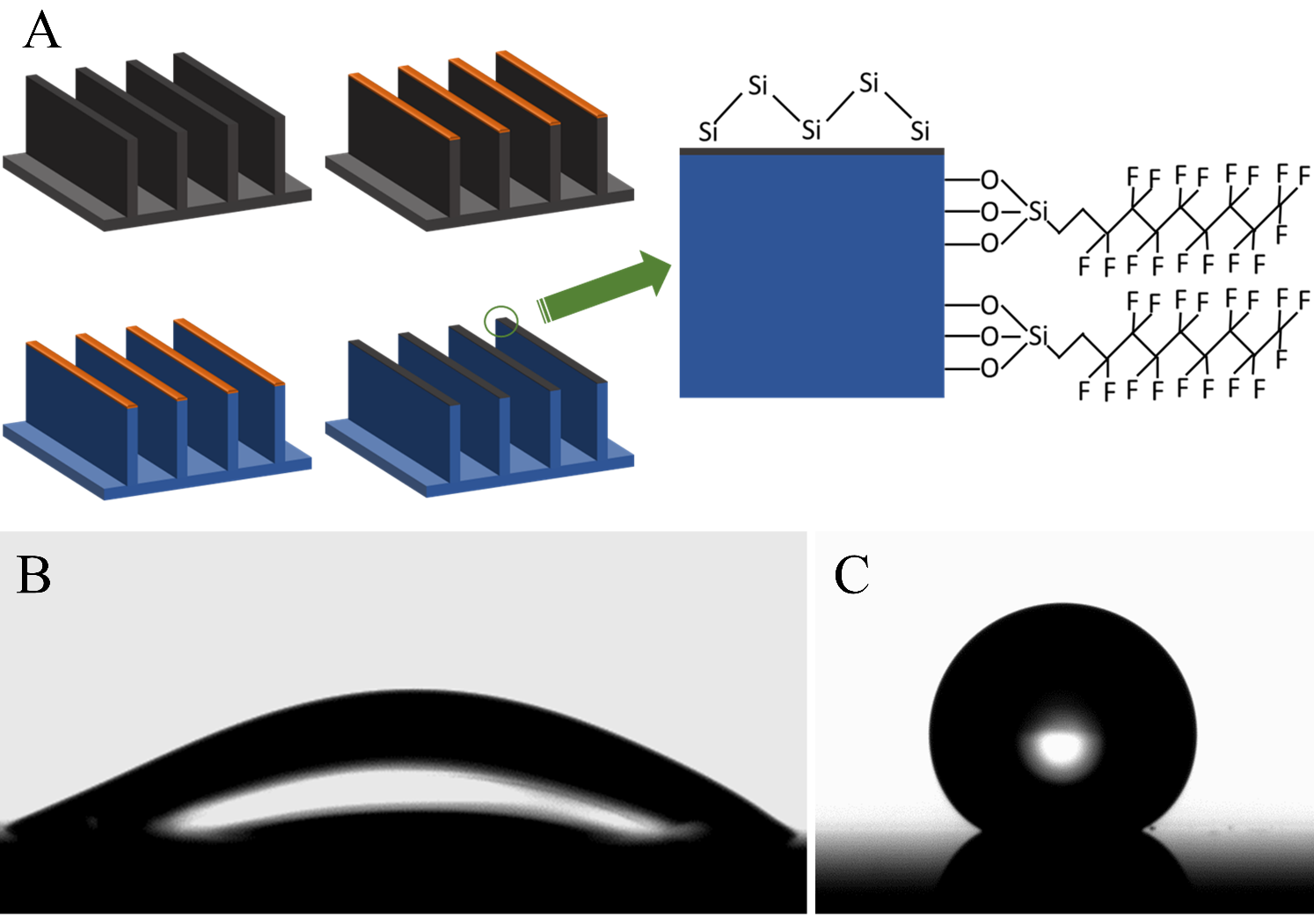


Supplementary Figure 1. (A) Asymmetric modification of silicon micropillar templates. The top of the silicon micropillar was covered with a thin film of photoresist (KMP CP4800). The silicon pillar template was then modified in FAS atmosphere, and then the thin layer of photoresist on the top of the pillar was washed away to obtain an asymmetric wettable template with a hydrophilic top and hydrophobic sidewalls. Gray, orange and blue represent the silicon micropillar, photoresist and FAS, respectively; (B) Contact angle of the unmodified template is 42.7 ± 0.8°; (C) Contact angle of the asymmetrically modified silicon micropillar template is 144.4 ± 2.9°.


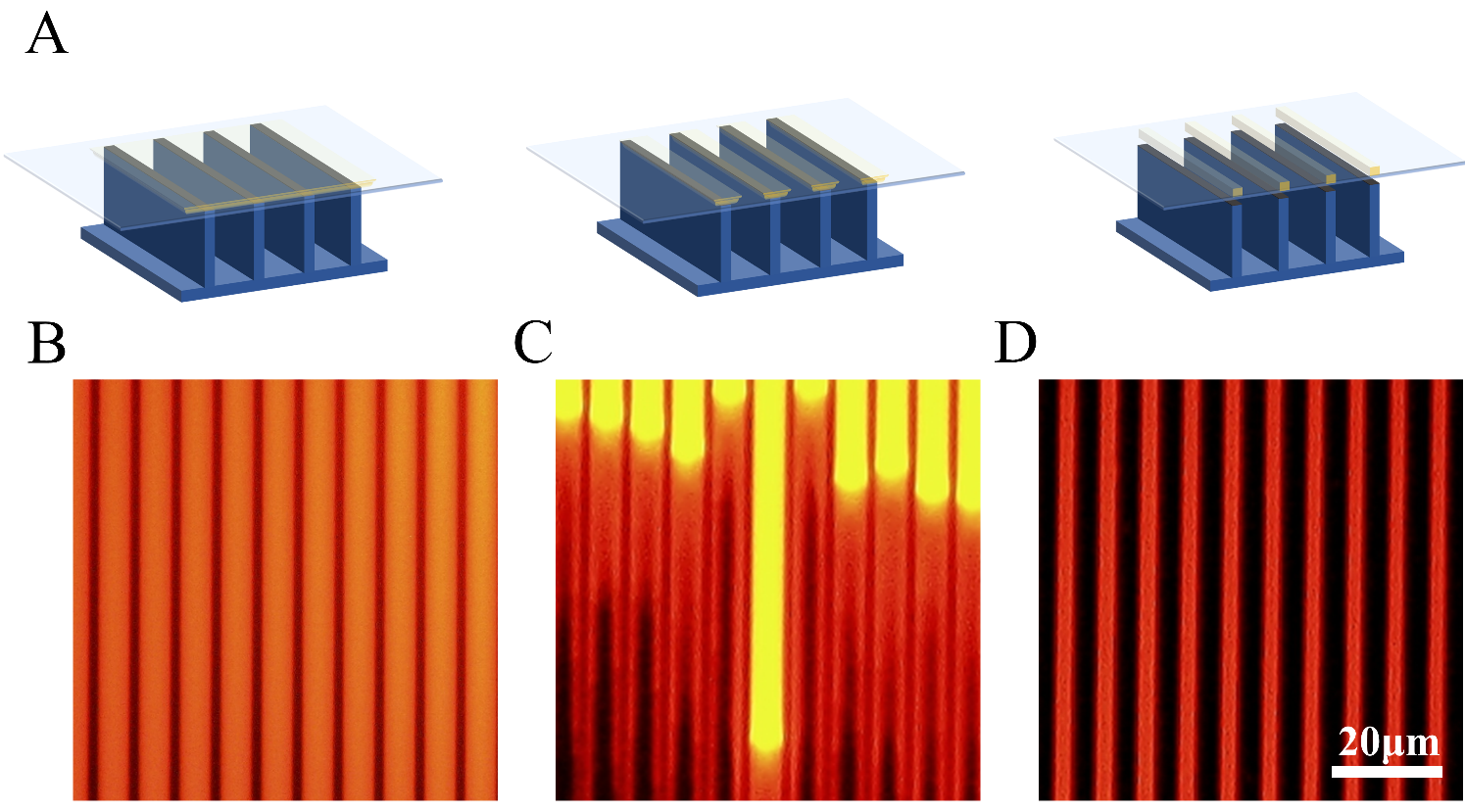


Supplementary Figure 2. (A) Schematic diagram of the CBMA process. The sandwich structure is a polished quartz glass sheet, polymer/AIE molecules solution and asymmetric wettability template in order from top to bottom. With the slow evaporation of the solution, the liquid film is gradually split into capillary bridges and eventually forms a precisely aligned array of polymer/AIE molecular microfilaments; (B) Dark field photograph of the continuous liquid film in the sandwich structure; (C) Dark field photograph of the capillary bridges formed by breaking the liquid film as the solution evaporates; (D) Dark field photograph of the precisely aligned array of microfilaments eventually formed at this location.


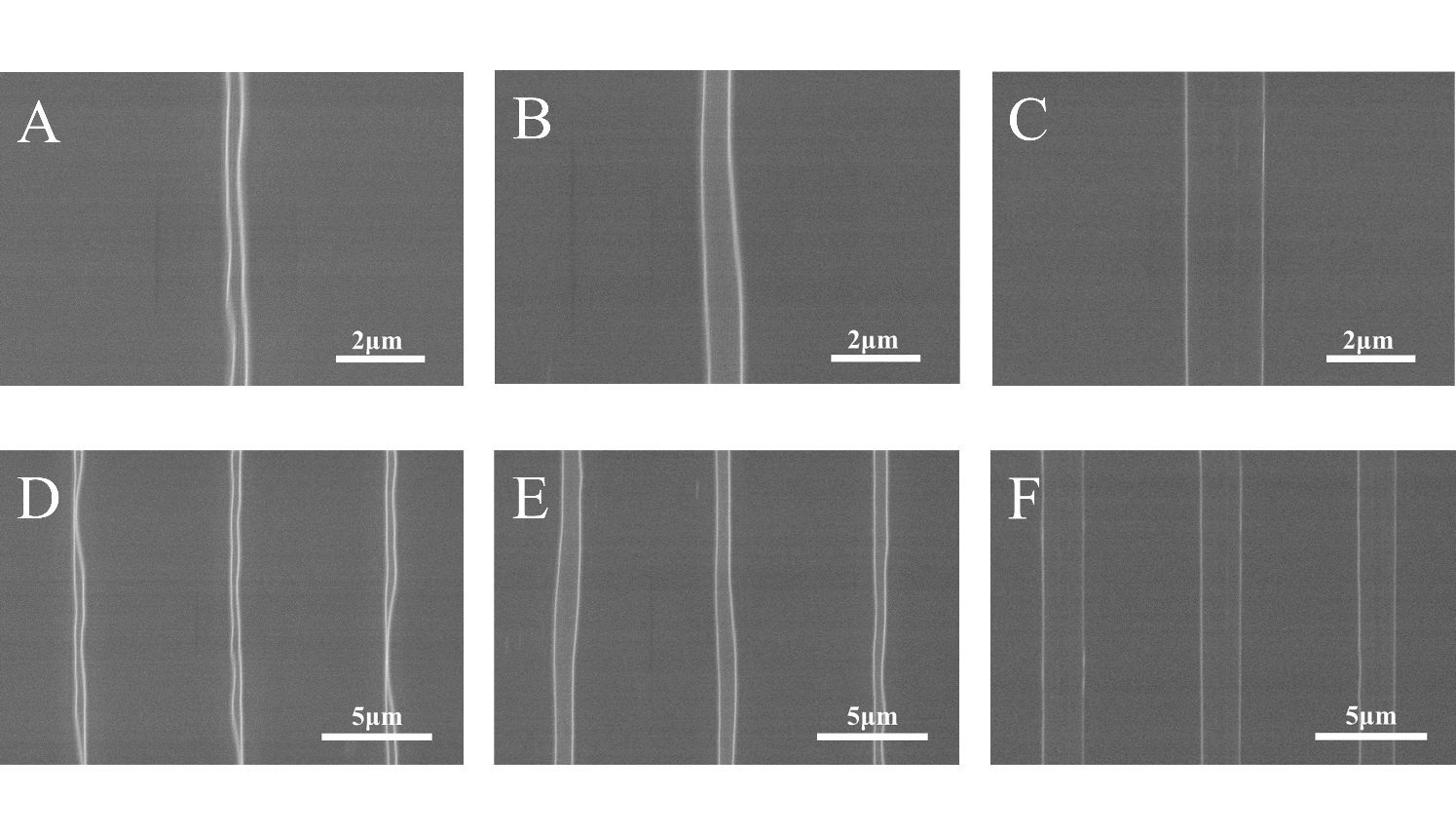


Supplementary Figure 3. Micron filaments become more and more full as the concentration of polymer/AIE molecule solution increases, and the prepared micron filaments have flat edges and are very neatly arranged when the solution concentration reaches 10 g/L. (A, D) SEM images of microfilament arrays prepared with 1 g/L solution; (B, E) SEM images of microfilament arrays prepared with 5 g/L solutions; (C, F) SEM images of microfilament arrays prepared with 10 g/L solution.


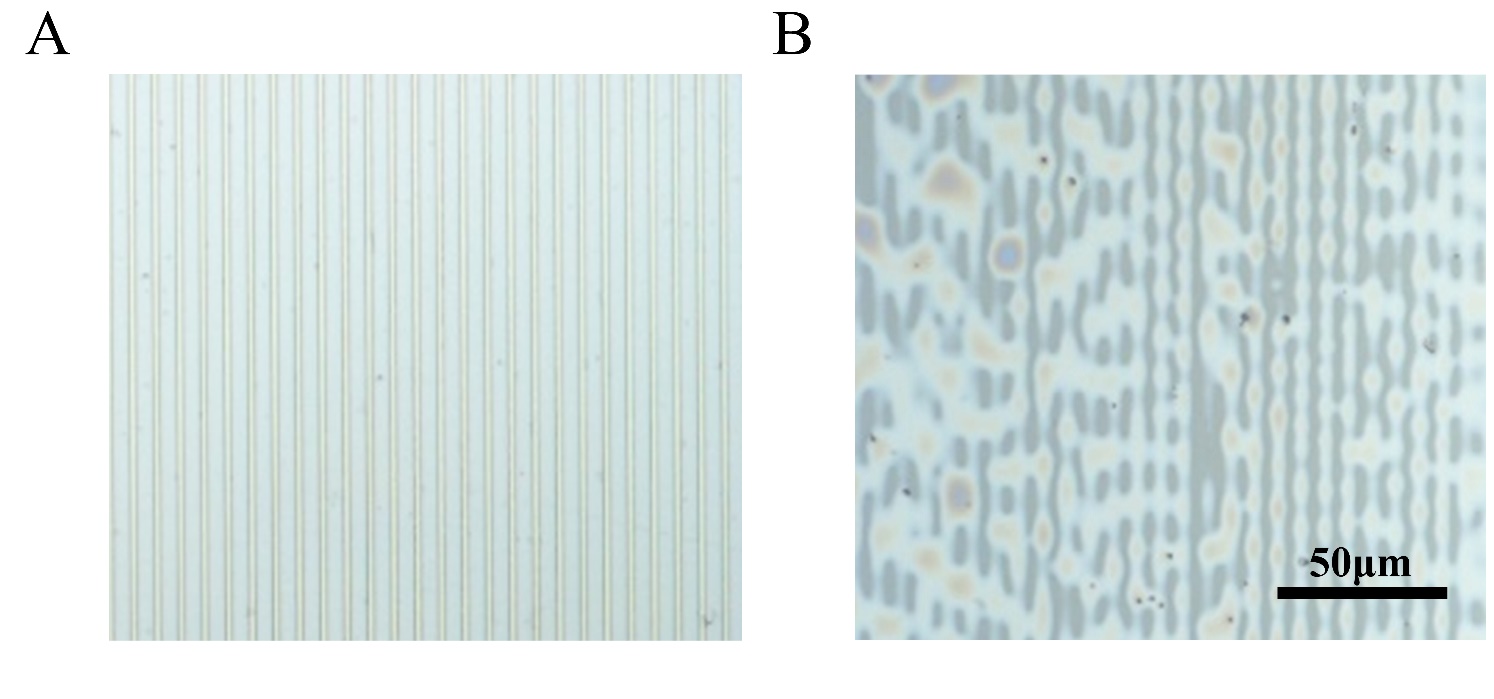


Supplementary Figure 4. (A) Bright-field photograph of polymer/AIE molecular microfilament arrays prepared using asymmetrically modified templates; (B) Highly aligned microfilament arrays that could not be prepared using unmodified templates.


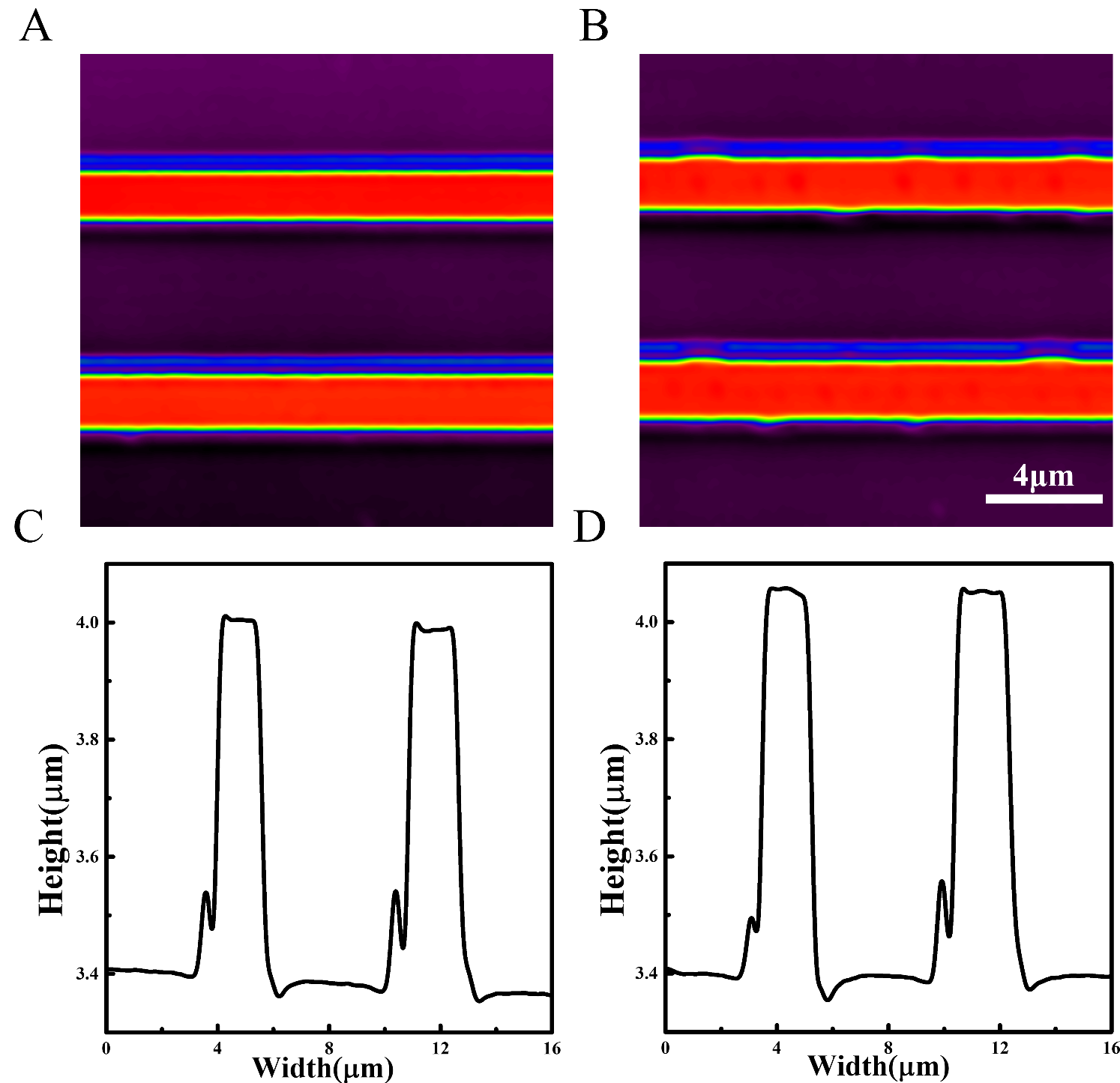


Supplementary Figure 5. (A) Confocal microscope image of PS/TPMN microfilament array in air atmosphere; (B) Confocal microscope image of PS/TPMN microfilament array in acetone vapor at 300000 ppm; (C) Cross-sectional confocal microscope images of PS/TPMN microfilament array in air atmosphere; (D) Cross-sectional confocal microscope images of PS/TPMN microfilament array in acetone vapor at 300000 ppm. The width and height of the dissolved one-dimensional structure become larger by 7.18 ± 1.17% and 8.66 ± 0.87%, respectively.


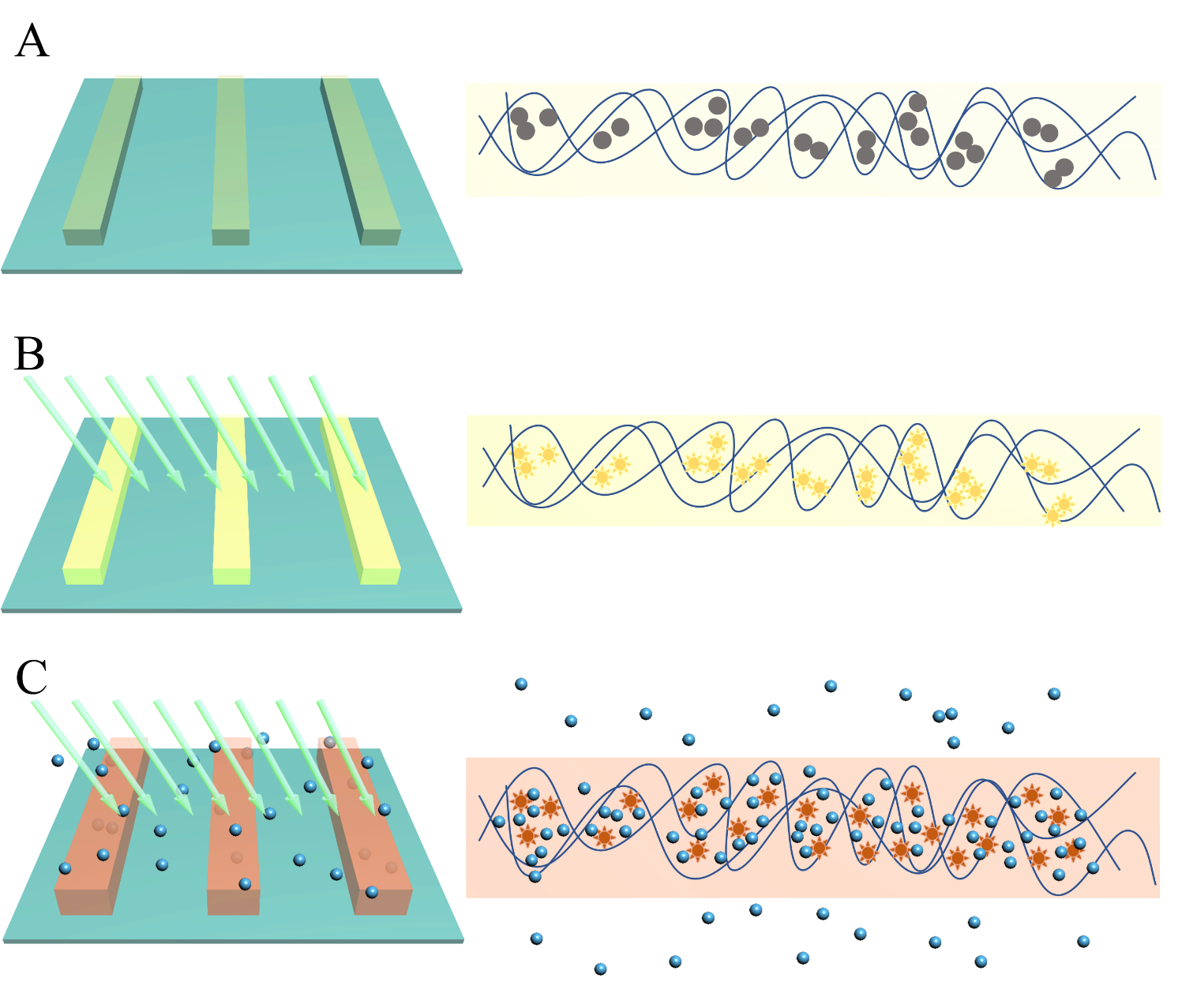


Supplementary Figure 6. Schematic of sensing mechanism. (A) AIE molecules are tightly entangled in polymer chains but the microfilament array does not emit light in the absence of excitation light; (B) The microfilament array emits bright yellow light under the excitation light of 480nm; (C) When acetone vapor is blown in, the acetone molecules get between the polymer chains, microfilament swelling occurring and making the distance between the AIE molecules increase, which leads to a decrease in fluorescence intensity. Meanwhile, the contact between acetone molecules and AIE molecules is affected by the solvation effect, which leads to a change in the luminescence color of AIE molecules from yellow to red.
